# Patient and Partner Sexual Outcomes After Transperineal Laser Ablation for Benign Prostatic Hyperplasia: A 12-Month Longitudinal Study

**DOI:** 10.3390/jcm15218599

**Published:** 2026-11-04

**Authors:** Yusuf Ozlulerden, Duran Duzgun, Sinan Celen, Mesut Berkan Duran, Kursat Kucuker, Aykut Akinci, Aykut Baser, Ahmet Baki Yagci

**Affiliations:** 1 Department of Urology, Faculty of Medicine, Pamukkale University, Denizli 20070, Turkey; sinancelen@hotmail.com (S.C.); kkucuker@pau.edu.tr (K.K.); aykut-akinci@hotmail.com (A.A.); 2 Department of Urology, Kutahya Health Sciences University Kutahya City Hospital, Kutahya 43100, Turkey; duranduzgun49@gmail.com; 3 Department of Urology, Faculty of Medicine, Bandirma Onyedi Eylul University, Balikesir 10200, Turkey; abaser@bandirma.edu.tr; 4 Department of Radiology, Faculty of Medicine, Pamukkale University, Denizli 20070, Turkey; byagci@pau.edu.tr

**Keywords:** transperineal laser ablation, benign prostatic hyperplasia, erectile dysfunction, ejaculatory dysfunction, partner sexual function, minimally invasive surgical therapy

## Abstract

**Background:** Preservation of sexual function has become a central goal in the surgical management of benign prostatic hyperplasia (BPH)/lower urinary tract symptoms (LUTS), yet the impact of treatment on the patient’s partner has rarely been examined. Transperineal laser ablation (TPLA) is an emerging, explicitly ejaculation-sparing technique with a favorable reported sexual function profile. **Objective:** In this retrospective, single-center, single-arm cohort study, we aimed to longitudinally evaluate erectile function, ejaculatory function and bother, and partner sexual function from the first postoperative week to 12 months after TPLA. **Materials and Methods:** This retrospective study was based on prospectively collected outcome data from 36 consecutive sexually active men who underwent ultrasound-guided TPLA (SoracteLite™/EchoLaser™) for moderate-to-severe LUTS/BPH, with assessments at postoperative weeks 1 and 2 and months 1, 3, 6, 9, and 12. Erectile function (IIEF-5), ejaculatory function and bother (MSHQ-EjD), and partner sexual function (Female Sexual Function Index, FSFI) were assessed longitudinally alongside IPSS, Qmax, post-void residual volume, and prostate volume. **Results:** Median baseline IPSS was 31 and prostate volume was 70 mL. IIEF-5 declined transiently at weeks 1 and 2 (both *p* < 0.001), returned to baseline by month 1, and did not differ significantly from baseline thereafter, consistent with preservation of erectile function. MSHQ-EjD function scores likewise declined transiently in the early postoperative period before rising modestly above baseline from month 3 onward (all *p* < 0.001, months 3–12), with a parallel change in the bother domain over the same period. Partner FSFI scores followed a similar temporal pattern, declining transiently during the first postoperative month, normalizing by month 3, and was slightly higher than baseline at month 12 (*p* < 0.05). IPSS improved from 31 to 8 and prostate volume decreased by a median of 52.5% at 12 months (both *p* < 0.001). No Clavien–Dindo grade ≥II complication occurred. **Conclusions:** In this retrospective single-arm cohort, TPLA was followed by substantial relief of severe LUTS. The findings are compatible with preservation of erectile function and a small increase in ejaculatory function scores, with partner sexual function following a parallel, non-causal trajectory. In the absence of a comparator group, these later changes cannot be attributed to TPLA itself and may partly reflect urinary symptom relief, medication changes, regression to the mean, or expectation effects. Controlled studies incorporating partner-reported outcomes are needed to determine whether TPLA confers couple-level sexual benefit.

## 1. Introduction

Benign prostatic hyperplasia (BPH) is one of the most prevalent urological conditions in the aging male, with up to 50% of men in their fifties developing clinically significant lower urinary tract symptoms (LUTS) [1,2]. Beyond its impact on voiding function, BPH exerts a considerable burden on male sexual health. In the Multinational Survey of the Aging Male (MSAM-7), which included more than 12,000 men aged 50–80 years, the prevalence of erectile dysfunction and ejaculatory disorders among sexually active men was 49% and 46%, respectively, and the presence and severity of LUTS were independent risk factors for sexual dysfunction regardless of age or comorbidities [3]. Similar associations between LUTS severity and erectile dysfunction have been reported in men with moderate-to-severe symptomatic BPH [4].

Although transurethral resection of the prostate (TURP) remains the gold standard surgical intervention, it is associated with de novo erectile dysfunction in 3–32% and ejaculatory dysfunction in 53–72% of patients [5]. As preserving sexual function has become a primary goal in contemporary BPH management, interest in minimally invasive surgical therapies (MISTs) with favorable sexual outcome profiles has grown substantially [6,7].

Among these emerging modalities, transperineal laser ablation (TPLA) has attracted particular attention as an explicitly ejaculation-sparing approach. Using the SoracteLite™/EchoLaser™ system, TPLA induces coagulative necrosis of prostatic tissue through transperineally inserted laser fibers under ultrasound guidance, without transurethral resection, thereby preserving the anatomical structures critical for antegrade ejaculation and erectile function [8,9]. Prospective studies have consistently demonstrated significant improvements in IPSS, maximum urinary flow rate (Qmax), and QoL following TPLA, with antegrade ejaculation preserved in the vast majority of sexually active patients and erectile function remaining stable throughout follow-up [9,10,11,12]. In the only randomized trial comparing TPLA with TURP, 96% of TPLA-treated patients maintained antegrade ejaculation at one month versus only 28% in the TURP group [13]. More recently, the first randomized trial comparing TPLA with convective water vapor ablation demonstrated that both techniques preserve erectile function and improve ejaculatory scores at six months without a statistically significant difference between groups [14].

Despite these encouraging results, important gaps remain. First, the TPLA literature has focused almost exclusively on male-reported outcomes, primarily IIEF scores and the binary preservation of antegrade ejaculation, whereas the sexual function of the partner has not been systematically assessed [9,10,11,12,13,14]. Because sexual function is inherently relational, and because both urinary symptoms and their treatment may affect the couple’s sexual life, partner-reported outcomes are needed to provide a complete picture of the sexual consequences of BPH treatment. Second, most studies have assessed sexual function at 3, 6, or 12 months, so the early postoperative period, when transient changes related to edema and discomfort are most likely, remains poorly characterized. Third, ejaculatory bother has rarely been reported separately from ejaculatory function, although it may better reflect the patient’s own perception of change [14].

Therefore, the present study aimed to longitudinally evaluate erectile function, ejaculatory function and bother, and partner sexual function from the first postoperative week to 12 months after TPLA in sexually active men with LUTS/BPH. We hypothesized that, after a possible transient early decline, erectile and ejaculatory function would be preserved during 12 months of follow-up, and that partner sexual function would follow a similar trajectory without sustained deterioration.

## 2. Materials and Methods

### 2.1. Study Design and Patient Population

**Figure 1:**
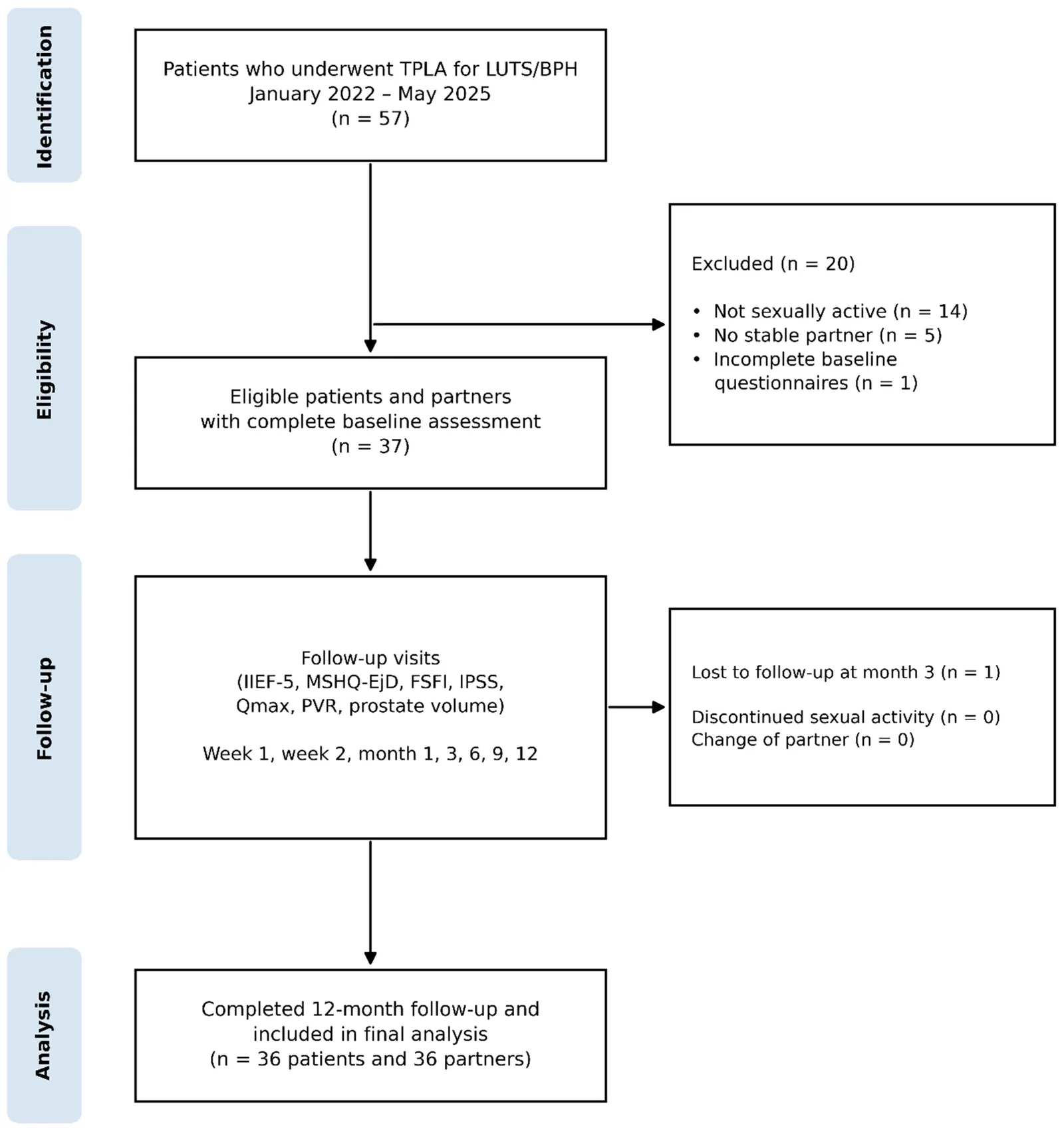
STROBE flow diagram of patient selection and follow-up. TPLA, transperineal laser ablation; LUTS, lower urinary tract symptoms; BPH, benign prostatic hyperplasia; IIEF-5, International Index of Erectile Function-5; MSHQ-EjD, Male Sexual Health Questionnaire–Ejaculatory Dysfunction; FSFI, Female Sexual Function Index; IPSS, International Prostate Symptom Score; Qmax, maximum urinary flow rate; PVR, post-void residual volume.

This retrospective, single-center study was based on prospectively collected clinical data from 36 consecutive sexually active male patients who underwent ultrasound-guided TPLA for LUTS secondary to BPH between 1 January 2022 and 1 May 2025. Although the overall study design and analysis were retrospective, all outcome data—including patient and partner questionnaires—were collected prospectively at each scheduled clinical visit as part of a standardized routine follow-up protocol and recorded contemporaneously in patients’ clinical files by a resident not involved in the procedure, with no data reconstructed from medical records. Data completeness was verified by cross-referencing clinical records. Patients with complete follow-up data through 12 months were included in the analysis (Figure 1). The study was conducted in accordance with the Declaration of Helsinki and was approved by the Pamukkale University Non-Interventional Clinical Research Ethics Committee on 6 July 2026 (Approval No: E.898402) for the retrospective analysis of these routinely collected clinical data; the committee waived the requirement for formal research-specific informed consent from both patients and their partners, given the routine clinical nature of data collection.

Inclusion criteria were: male sex; age ≥ 45 years; moderate-to-severe LUTS with an International Prostate Symptom Score (IPSS) ≥ 8; prostate volume ≥ 30 mL as assessed by transrectal ultrasound (TRUS) or multiparametric MRI (mpMRI); Qmax ≤ 15 mL/s; serum PSA < 10 ng/mL, or PSA ≥ 10 ng/mL with a previously negative mpMRI or prostate biopsy; failure of or intolerance to medical therapy, or strong patient preference for preservation of antegrade ejaculation; and confirmed sexual activity with a stable female partner at the time of enrollment, defined as having engaged in sexual intercourse at least once per month during the preceding three months.

Exclusion criteria included a history of surgery involving the prostate, bladder neck, or urethra, clinical suspicion or history of prostate and bladder cancer, active urinary tract infection, history of pelvic radiotherapy, known neurological disorders affecting bladder function (e.g., multiple sclerosis, Parkinson’s disease, spinal cord injury), bladder stones, large intravesical prostatic protrusion (>1.5 cm).

### 2.2. Partner Assessment

Female partners’ sexual function was assessed using the validated Turkish version of the 19-item Female Sexual Function Index (FSFI), which evaluates six domains (desire, arousal, lubrication, orgasm, satisfaction, and pain), yielding a total score of 2–36; a total score ≤ 26.55 indicates female sexual dysfunction [15]. The questionnaire was self-administered by the partner independently of the patient in a separate room at baseline and at each follow-up visit. Questionnaires were distributed and collected by a resident not involved in the procedure. The same partner was assessed at all time points; patients whose partner changed during follow-up were not included.

### 2.3. Surgical Technique

All procedures were performed by a single surgeon who had completed TPLA training and proctoring. All TPLA procedures were performed using the SoracteLite™/EchoLaser™ system (Elesta S.p.A., Florence, Italy), a continuous-wave multi-source diode laser operating at 1064 nm. Patients were placed in the lithotomy position under local perineal anesthesia (lidocaine 2%, 20 mL) combined with an ultrasound-guided periprostatic nerve block. A transurethral catheter was inserted prior to the procedure to enable urethral identification during real-time transrectal ultrasound monitoring.

Under biplanar transrectal ultrasound guidance, one or two 21-gauge introducer needles were placed transperineally into the prostate, parallel to the long axis of the gland. Needle placement was assisted by the EchoLaser Smart Interface (ESI) planning software, which projects predefined safety margins onto the ultrasound image. Mandatory safety distances were maintained from the urethra (≥8 mm), prostatic capsule (≥10 mm), and bladder neck (≥15 mm) to minimize the risk of urethral injury and ejaculatory dysfunction. Following needle placement, 300-μm optical fibers were introduced and ablation was performed at a fixed power of 3–5 W, delivering up to 1800 J per fiber. Where prostate dimensions required, a pull-back maneuver with re-illumination was performed. During and prior to the TPLA procedure, continuous cold saline irrigation (+4 °C) was applied to reduce the thermal effect of the laser energy. All patients received standard antibiotic prophylaxis perioperatively and a short course of corticosteroids to reduce postoperative edema.

### 2.4. Outcome Measures

The primary endpoints of this study were changes in erectile and ejaculatory function, assessed using the International Index of Erectile Function 5 (IIEF-5) and the Male Sexual Health Questionnaire—Ejaculatory Dysfunction short form (MSHQ-EjD), including its bother domain, at each follow-up visit. For the bother item, higher scores indicate less bother. Female partners’ sexual function was additionally evaluated using the Female Sexual Function Index (FSFI). Secondary endpoints included changes in IPSS, QoL score, Qmax, post-void residual volume (PVR), prostate volume, and PSA at postoperative weeks 1 and 2, and months 1, 3, 6, 9, and 12. Sexual activity during the preceding period was confirmed at each visit. Intraoperative parameters including total energy applied, number of fibers used, Pain intensity with VAS score and procedure duration were recorded. Comorbidity burden was assessed using the Charlson Comorbidity Index (CCI), and complications were graded according to the Clavien–Dindo classification. To assess clinical relevance, changes were interpreted against minimal clinically important difference (MCID) thresholds: 4 points for IIEF-5, based on the anchor-based MCID of the IIEF erectile function domain [16], and 4.8 points for FSFI total score [17]. As no MCID has been published for the MSHQ-EjD, changes in this instrument were interpreted descriptively.

### 2.5. Statistical Analysis

Given the modest sample size and the non-normal distribution of most variables (Shapiro–Wilk test), continuous variables are presented as median (interquartile range, IQR) and categorical variables as frequencies and percentages. Analyses were performed on a complete-case basis; data from the one patient lost to follow-up at month 3 were excluded from all analyses, and no imputation was performed. As there were no missing values in the analyzed cohort, all comparisons included the same 36 patients at every time point. For each outcome, changes over time across all eight time points were first assessed using the Friedman test, with Kendall’s W as a measure of effect size. When the Friedman test was significant, post hoc comparisons between baseline and each follow-up time point were performed using the Wilcoxon signed-rank test. Zero differences were excluded, tied absolute differences were assigned average ranks, and *p*-values were obtained from the normal approximation with correction for ties. To account for multiple comparisons, *p*-values from the seven post hoc comparisons of each outcome measure were adjusted using the Holm method. Changes from baseline are presented as Hodges–Lehmann estimates of the median within-patient change with 95% confidence intervals (CI). As a sensitivity analysis, each outcome was also analyzed using a linear mixed-effects model with time as a categorical fixed effect (baseline as reference) and a random intercept for each patient, with Holm adjustment of the seven comparisons against baseline. Exploratory correlation analyses between changes in prostate volume and changes in sexual-function scores (IIEF-5, MSHQ-EjD, MSHQ-bother, and FSFI), and between changes in IIEF-5 and MSHQ-bother scores, were performed at each follow-up time point using Spearman’s rank correlation coefficient (rho). Given the number of tests performed, *p*-values were adjusted using the Holm method, and these analyses were considered hypothesis-generating only. As all consecutive eligible patients treated during the study period were included, no a priori sample size calculation was performed. Instead, a sensitivity power analysis was conducted to determine the minimum detectable within-subject change with 36 paired observations, a two-sided α of 0.05 (and 0.05/7 to reflect Holm adjustment), and 80% power, adjusted for the asymptotic relative efficiency of the Wilcoxon signed-rank test (0.955).

All tests were two-tailed, and an adjusted *p*-value < 0.05 was considered statistically significant. Statistical analyses were performed using SPSS Statistics version 27.0 (IBM Corp., Armonk, NY, USA); Hodges–Lehmann estimates and the mixed-effects models were calculated using Python version 3.9.7 (SciPy version 1.7.3 and statsmodels version 0.13.0 libraries).

## 3. Results

### 3.1. Patient Population and Baseline Characteristics

During the study period, 57 patients underwent TPLA for LUTS/BPH. Of these, 20 were excluded (not sexually active, *n* = 14; no stable partner, *n* = 5; incomplete baseline questionnaires, *n* = 1). Of the 37 eligible patients, 1 was lost to follow-up at month 3, leaving 36 patients and their partners for analysis (Figure 1). All 36 patients and partners completed the questionnaires at every scheduled visit, with no missing data, and no patient discontinued sexual activity or changed partner during follow-up. All patients and partners reported sexual activity at every follow-up visit, including the first and second postoperative weeks. The median age was 59 years (IQR 9.75) and the baseline median prostate volume was 70 mL (IQR 19.5) while the median PSA was 3.75 ng/mL (IQR 1.89). One patient had a baseline PSA ≥ 10 ng/mL and was included after a negative mpMRI. At baseline, the median IPSS was 31 (IQR 6), QoL score 4 (IQR 1), Qmax 7.0 mL/s (IQR 2.0), and PVR of 280 mL (IQR 167.5). Regarding baseline sexual function, the median IIEF-5 score was 16 (IQR 5); the median MSHQ-EjD score was 11 (IQR 3) with a median bother-domain score of 3 (IQR 1); and the median partner FSFI score was 23 (IQR 9.75). The median Charlson Comorbidity Index was 2 (IQR 2). Of the 36 patients, 7 (19.4%) were treatment-naive and chose TPLA because of a strong preference for preservation of antegrade ejaculation, 20 (55.6%) had experienced failure of medical therapy, and 9 (25%) had discontinued medical therapy because of intolerance. Baseline characteristics are summarized in Table 1.

### 3.2. Perioperative Outcomes

TPLA was successfully completed in all patients. The median procedure duration was 27.5 minutes (IQR 13), while the median total laser energy delivered was 7200 J (IQR 3600), corresponding to a median (IQR) energy density of 81.35 (26.11) J/mL. A median of 2 (IQR 1) optical fibers (range 1–2) were used per procedure. The procedure was well tolerated under local anesthesia, with a median intraoperative VAS pain score of 3 (IQR 2). Perioperative parameters are summarized in Table 2.

### 3.3. Sexual Outcomes

All four sexual-function scores changed significantly over time (Friedman test, all *p* < 0.001; Kendall’s W: IIEF-5 0.69, MSHQ-EjD 0.92, MSHQ-bother 0.77, FSFI 0.72), with a transient decline in the immediate postoperative period (weeks 1 and 2) followed by a return to baseline.

**Figure 2:**
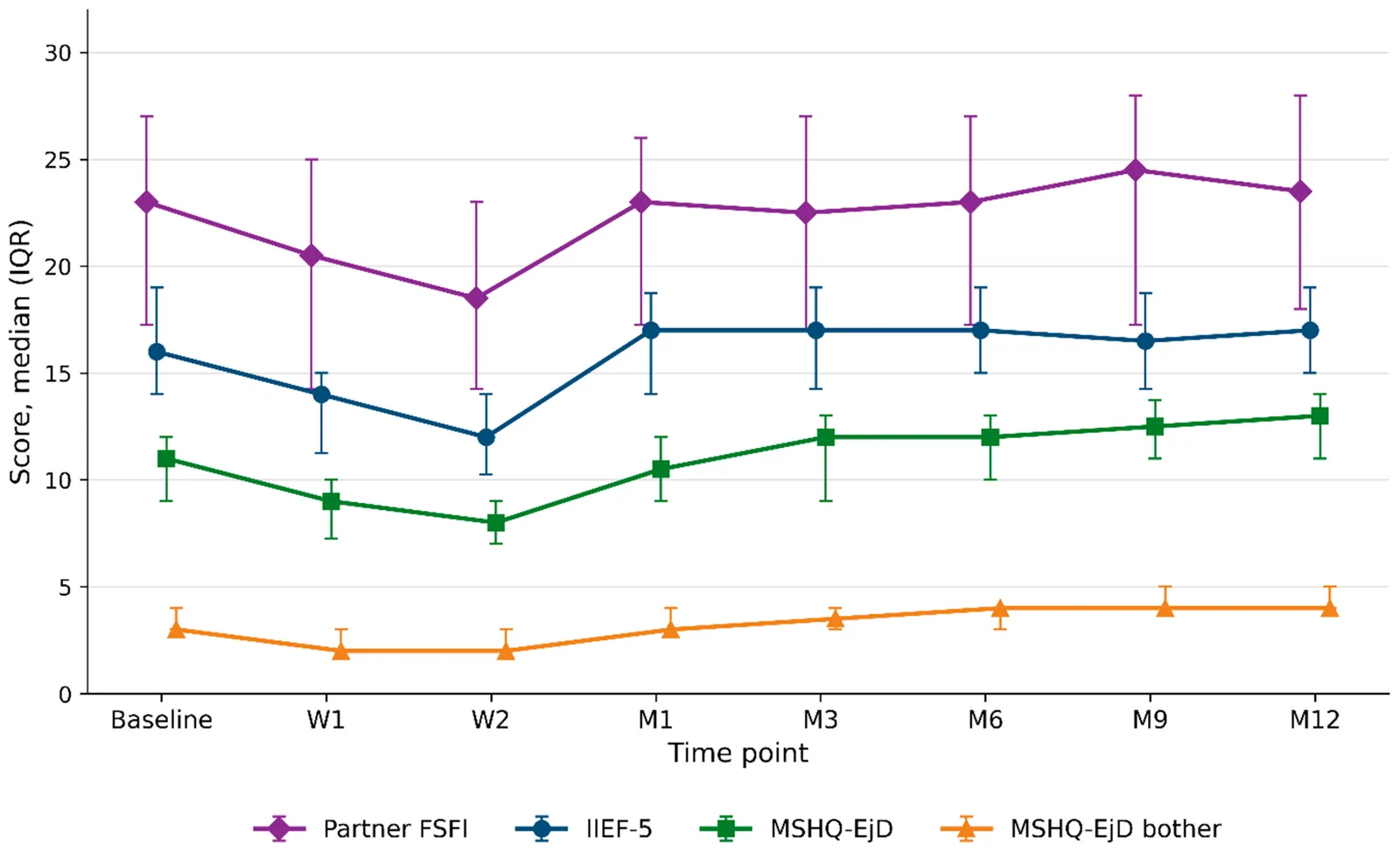
Patient and partner sexual function scores over 12 months after transperineal laser ablation (TPLA) of the prostate. Points show median scores and error bars the interquartile range (IQR) for the International Index of Erectile Function-5 (IIEF-5), the Male Sexual Health Questionnaire–Ejaculatory Dysfunction short form (MSHQ-EjD) function and bother domains, and the partner-reported Female Sexual Function Index (FSFI), at baseline, week 1 (W1), week 2 (W2), and months 1, 3, 6, 9, and 12 (M1–M12) (*n* = 36). For the MSHQ-EjD bother item, higher scores indicate less bother. All scores declined transiently during the first two postoperative weeks and returned to around baseline by month 1–3; changes from baseline with 95% confidence intervals are reported in the text.

The IIEF-5 score decreased significantly from baseline at postoperative week 1 (median change −3.0 points, 95% CI −3.0 to −2.5) and week 2 (−4.0 points, 95% CI −4.5 to −3.5; both adjusted *p* < 0.001). By month 1, the IIEF-5 score had returned to baseline (0.0 points, 95% CI 0.0 to 0.0; adjusted *p* = 0.763) and it did not differ significantly from baseline at any subsequent time point (month 12: 0.5 points, 95% CI 0.0 to 0.5; adjusted *p* = 0.053) (Figure 2).

The MSHQ-EjD score likewise decreased significantly in the early postoperative period (week 1: −2.0 points, 95% CI −2.5 to −2.0; week 2: −3.0 points, 95% CI −3.0 to −2.5; both adjusted *p* < 0.001) and remained slightly below baseline at month 1 (0.0 points, 95% CI −0.5 to 0.0, adjusted *p* = 0.008). From month 3 onward, the MSHQ-EjD score was modestly higher than baseline (month 3: 1.0 point, 95% CI 0.5 to 1.0; month 6: 1.0 point, 95% CI 1.0 to 1.5; month 9: 1.5 points, 95% CI 1.0 to 1.5; month 12: 2.0 points, 95% CI 1.5 to 2.0; all adjusted *p* < 0.001). A parallel pattern was observed for the MSHQ-bother domain, with a significant early decrease at week 1 (−1.0 point, 95% CI −1.0 to −0.5) and week 2 (−1.0 point, 95% CI −1.0 to −1.0; both adjusted *p* < 0.001), values close to baseline at month 1 (0.0 points, 95% CI 0.0 to 0.0; adjusted *p* = 0.029) and month 3 (0.0 points, 95% CI 0.0 to 0.5; adjusted *p* = 0.058), and higher bother scores (i.e., less bother) at month 6 (0.5 points, 95% CI 0.0 to 0.5; adjusted *p* = 0.009), month 9 (1.0 point, 95% CI 0.5 to 1.0), and month 12 (1.0 point, 95% CI 1.0 to 1.0; both adjusted *p* < 0.001) (Figure 2).

Partners’ FSFI scores decreased significantly at week 1 (−2.5 points, 95% CI −3.0 to −2.0) and week 2 (−3.5 points, 95% CI −4.0 to −3.0; both adjusted *p* < 0.001) and remained slightly below baseline at month 1 (−0.5 points, 95% CI −0.5 to 0.0; adjusted *p* = 0.007). By month 3, scores had returned to baseline (0.0 points, 95% CI 0.0 to 0.0; adjusted *p* = 1.000). Thereafter, FSFI scores were slightly higher than baseline at month 12 (0.5 points, 95% CI 0.5 to 1.0; adjusted *p* = 0.001); the small increase at month 9 (0.5 points, 95% CI 0.0 to 0.5; adjusted *p* = 0.038) was not confirmed in the mixed-effects sensitivity analysis, and no significant difference was observed at month 6 (adjusted *p* = 0.063) (Figure 2).

Compared with MCID thresholds, the decline in IIEF-5 at week 2 (median change −4.0 points) reached the MCID of 4 points, whereas the decline in partner FSFI at week 2 (−3.5 points) remained below the MCID of 4.8 points. All changes from month 1 onward were below the MCID for IIEF-5 (≤0.5 points) and FSFI (≤0.5 points), and the increase in MSHQ-EjD at month 12 was 2.0 points on a 15-point scale.

In the sensitivity power analysis, the study could detect a standardized within-subject change of 0.49 (0.63 at the Holm-adjusted α). Given the observed standard deviation of within-patient FSFI changes (0.63–1.32 points), this corresponded to a minimum detectable change of 0.4–0.8 points, and the power to detect a 4.8-point change in FSFI (the MCID) exceeded 99% at all time points.

### 3.4. Urinary Functional Outcomes

All urinary parameters, prostate volume, and PSA changed significantly over time (Friedman test, all *p* < 0.001; Kendall’s W 0.94–0.99). IPSS improved progressively and significantly at every postoperative time point compared with baseline (all *p* < 0.001), decreasing from a baseline median of 31 (IQR 6) to 28 (IQR 4.75) at week 1, 26 (IQR 4.75) at week 2, 19 (IQR 4) at month 1, 15 (IQR 4) at month 3, 13 (IQR 3) at month 6, 10 (IQR 2) at month 9, and 8 (IQR 2) at month 12.

Prostate volume likewise decreased progressively and significantly throughout follow-up (all *p* < 0.001), from a baseline median of 70 mL (IQR 19.5) to 68 mL (IQR 19.25) at week 1, 65.5 mL (IQR 20) at week 2, 55 mL (IQR 17.25) at month 1, 45 mL (IQR 14) at month 3, 40 mL (IQR 14.25) at month 6, 37 mL (IQR 10.5) at month 9, and 32 mL (IQR 10.25) at month 12, corresponding to a median per-patient volume reduction of 52.5% from baseline to month 12.

Median serum PSA increased transiently from 3.75 ng/mL (IQR 1.89) at baseline to 4.70 ng/mL (IQR 2.55) at week 1 and 5.25 ng/mL (IQR 3.18) at week 2, consistent with ablation-related tissue necrosis, and decreased to 4.35 ng/mL (IQR 2.52) at month 1. Thereafter, PSA declined progressively to 3.50 ng/mL (IQR 1.95) at month 3, 2.90 ng/mL (IQR 1.38) at month 6, 2.65 ng/mL (IQR 1.70) at month 9, and 2.20 ng/mL (IQR 1.08) at month 12 (all *p* < 0.001 vs. baseline). At month 12, PSA was below baseline in all patients (median reduction 38%). No patient showed a PSA rise of ≥2 ng/mL above his lowest post-procedural value, and no patient required further evaluation for suspected prostate cancer during follow-up.

### 3.5. Correlation Analyses

In exploratory analyses, no statistically significant correlation was found between the reduction in prostate volume and the change in IIEF-5 score at any follow-up time point. Nominally significant correlations were observed between the reduction in prostate volume and the changes in MSHQ-EjD score (rho = 0.417, unadjusted *p* = 0.011) and partner FSFI score (rho = 0.355, unadjusted *p* = 0.034) at month 1, and between the changes in IIEF-5 and MSHQ-bother scores at week 2 (rho = 0.472, unadjusted *p* = 0.004). None of these correlations remained significant after Holm adjustment for multiple comparisons (adjusted *p* > 0.10 for all), and no other correlations were nominally significant.

### 3.6. Safety Outcomes

The procedure was well tolerated, with no major complications.

Among the 36 patients, dysuria occurred in 6 (16.7%) and transient perineal pain in 7 (19.4%), whereas no complications were observed in the remaining 23 patients (63.9%). All postoperative complications were managed conservatively, and no patient required surgical re-intervention or experienced Clavien–Dindo Grade II or higher complications. No cases of severe bleeding, urinary incontinence, urethral injury, or procedure-related hospitalization were recorded.

## 4. Discussion

As preservation of sexual function has become a key determinant in the management of BPH, evaluation of treatment outcomes should extend beyond the patient alone. To the best of our knowledge, this is the first study to assess sexual function in both men undergoing TPLA and their female partners. Consistent with our hypothesis, erectile and ejaculatory function scores were preserved after a transient early decline, and partner sexual function followed a similar trajectory. These observations suggest that TPLA is not associated with lasting deterioration in either male or partner sexual function. From a counseling perspective, these findings offer reassurance to couples considering TPLA, and comparative studies incorporating partner-centered outcomes may further define its role in preserving couple sexual well-being.

The early IIEF-5 dip during the first two postoperative weeks, resolving fully by month 1, is consistent with transient periprocedural edema, thermal inflammation around the neurovascular bundles, and confounding factors such as perineal discomfort, dysuria, and anxiety, rather than structural erectile injury particularly since mandatory capsular safety margins were respected in every case. This trajectory matches the broader TPLA literature: early prospective series reported stable outcomes without clinically relevant IIEF deterioration, including 12-month stability in one cohort and an unchanged total IIEF-15 score in another [9,12], and MRI-based studies confirmed preserved sexual function alongside substantial adenoma volume reduction [18]. A meta-analysis of 17 studies with 777 patients found no significant adverse effect of TPLA on IIEF-5 [19], and a more recent pool analysis restricted to six studies showed only a marginal, non-significant change at 6 months [20]. A MIST-wide meta-analysis similarly found statistically significant pooled IIEF gains only with Rezum and UroLift, with TPLA, prostatic artery embolization, iTIND, and Optilume classified as erectile-neutral [21], a conclusion echoed by a recent narrative review reporting non-significant IIEF-5 changes at 6–12 months in most cohorts [22]. In our series, the small numerical increase in IIEF-5 at 12 months (0.5 points, well below the MCID of 4 points) did not remain significant after adjustment for multiple comparisons or in the mixed-effects sensitivity analysis, supporting preservation rather than improvement of erectile function. This aligns with the broader view that TPLA’s principal erectile advantage over TURP lies in avoidance of de novo dysfunction rather than functional enhancement—a distinction with direct relevance for preoperative counseling, since patients should be informed that the treatment aim is protection of existing erectile capacity rather than its improvement. Notably, the decline in IIEF-5 at week 2 reached the MCID, indicating a transient but clinically perceptible impairment that should be discussed during preoperative counseling. This profile is underpinned by TPLA’s anatomical and technical characteristics: targeted coagulative necrosis confined to the transition zone, delivered under real-time ultrasound guidance with predefined safety margins from the urethra, capsule, and bladder neck, without the resection cavity created by TURP [18]. Contemporary reviews accordingly describe TPLA as a highly selective, organ-sparing therapy of relevance for men who prioritize sexual preservation [20,23,24].

The modest increase in MSHQ-EjD function scores above baseline from month 3 onward, together with a parallel reduction in ejaculatory bother, is a more distinctive finding, though not without precedent. As no MCID has been established for the MSHQ-EjD, the clinical relevance of this 2-point increase on a 15-point scale cannot be determined with certainty. Antegrade ejaculation was preserved in the large majority of patients across prospective TPLA series, including 95.5% in one cohort and all sexually active men in another 100-patient series [9,11], and van Kollenburg et al. reported preservation of antegrade ejaculation in 85% of evaluable patients [12]. A pooled analysis found an aggregate improvement in the MSHQ-EjD function domain of 2.64 points, with the largest single-study effect showing an increase from 4 to 12 points at 3 years—a trajectory closely resembling the late increase observed in our cohort [20]. A separate systematic review similarly identified TPLA as producing MSHQ improvements of 2.8–3.8 points, among the most favorable ejaculatory profiles of all MISTs evaluated [25]. In the randomized comparison against convective water vapor ablation, the MSHQ-EjD function score improved from 6 to 9.5 at 6 months in the TPLA group while the bother score fell from 3 to 1, an improvement that remained significant after adjustment for prostate volume, confirming that function and bother typically move in the same favorable direction after TPLA [14]. The parallel change we observed in both domains from month 3 onward is consistent with this pattern. However, in the absence of a control group, we cannot determine whether it reflects a direct treatment effect. Several factors, not all related to the direct effect of ablation, may account for this delayed change. First, resolution of severe obstructive and storage symptoms—reflected in our cohort’s high baseline IPSS of 31—may itself improve perceived ejaculatory quality and reduce associated bother, independent of any direct anatomical effect on the ejaculatory apparatus, a mechanism explicitly discussed in the fifth International Consultation on Sexual Medicine (ICSM), which links LUTS severity, urgency, and coital incontinence to diminished ejaculatory satisfaction [26]. Second, ablation-mediated reduction in prostatic inflammation may also attenuate a chronic-prostatitis-related contribution to acquired ejaculatory dysfunction [26]. Third, and importantly, discontinuation of alpha-blockers or 5-ARIs after symptomatic relief represents a potential confounder rather than a mechanism attributable to TPLA itself. Both drug classes are known to impair ejaculatory volume and force, and their withdrawal could produce an apparent improvement in ejaculatory function independent of any procedural effect, as described after water-vapor thermal therapy [21]. Because medication use and discontinuation were not systematically recorded in our cohort, we cannot estimate the extent to which this factor contributed to the observed change, and the late increase in MSHQ-EjD scores should not be attributed to TPLA itself. Future prospective studies should systematically document the use and discontinuation of alpha-blockers and 5-ARIs, as well as drug-related side effects, before and after the procedure. Fourth, regression to the mean and attention/expectation effects inherent to an unblinded, repeatedly assessed cohort may have contributed to higher scores at later visits. Consistent with the possibility that these changes are not driven by the extent of tissue ablation, our exploratory correlation analyses did not identify a robust association between prostate volume reduction and changes in sexual function after adjustment for multiple comparisons.

Compared with conventional transurethral surgery, the ejaculation-sparing advantage of TPLA is pronounced: in a randomized trial, MSHQ-EjD was unchanged after TPLA versus an approximate 30% reduction after TURP, with anejaculation in one TPLA patient versus 18 after TURP [13], and a comparative study likewise showed stable ejaculatory and erectile scores after TPLA despite greater flow improvement with TURP [27]. Across MISTs, pooled anejaculation rates range from 0% to 5%, still far below the 34–77% reported after TURP/HoLEP [21], while water-vapor therapy has shown rates around 10.8% [28]. No patient in our cohort reported anejaculation, which may relate to strict adherence to urethral, capsular, and bladder-neck safety margins [20].

Partner-reported outcomes remain an uncommon feature of the TPLA literature, which has focused predominantly on male IIEF and ejaculatory questionnaires or the binary preservation of antegrade ejaculation. Because sexual function is inherently relational, changes in male urinary symptoms, postoperative anxiety, and ejaculatory quality may also affect the partner, independent of any direct physiological effect of treatment on the partner herself. In our cohort, partner FSFI scores declined during the first postoperative month—plausibly related to the patient’s transient postoperative discomfort and the couple’s adaptation to these changes—returned to baseline by month 3, and were slightly higher than baseline at month 12, a trajectory that broadly paralleled the recovery and small subsequent increase in male ejaculatory function. This parallel trajectory suggests that the early postoperative period may affect the couple as a unit rather than the patient in isolation, and that recovery of partner sexual function may track the resolution of the patient’s own urinary and sexual symptoms. These findings are consistent with the ICSM’s expert-opinion-level recommendation that BPH/LUTS constitutes a shared stressor affecting the sexual well-being of both members of a couple, an area in which dyadic outcome data specific to surgical treatment remain scarce [26]. Nevertheless, the absolute FSFI changes were small and well below the MCID of 4.8 points, and partner-specific factors, such as age, menopausal status, hormonal treatment, gynecological conditions, medical comorbidities, baseline sexual distress, and relationship quality, were not assessed. Consequently, these findings should be interpreted as preservation and recovery of partner-reported sexual function paralleling the patient’s own recovery, rather than as evidence that TPLA directly improves female sexual function. Taken together, the parallel trajectories of male and partner scores are hypothesis-generating and justify prospective, controlled evaluation of dyadic outcomes after TPLA, with systematic collection of partner demographic, gynecological, and relational data to allow adjustment for these factors.

Urinary outcomes were similarly favorable, exceeding average pooled reductions reported in the literature [19], likely attributable to the high baseline symptom burden of our cohort. The median volume reduction of 52.5% at 12 months is at the upper end of that reported in previous TPLA series (approximately 32–41%) [9,29]. Patients treated with two fibers showed a numerically greater reduction than those treated with one fiber, suggesting a possible dose–response relationship, and the performance of all procedures by a single experienced surgeon may have contributed to consistent fiber positioning. However, differences in patient selection and volume measurement methods across studies (transrectal ultrasound vs. MRI) limit direct comparison. Consistent with recent proposals that MIST success should incorporate sexual preservation and quality of life alongside urinary improvement [23,24], our data are consistent with a function-preserving profile of TPLA and reinforce calls to report sexual outcomes alongside urinary parameters in future composite success definitions.

Strengths of this study include repeated sexual-function assessment from the first postoperative week to month 12, separate evaluation of erectile function, ejaculatory function, ejaculatory bother, and partner sexual function using validated instruments, and a standardized procedural technique. Limitations include the retrospective, single-center, single-arm design and modest sample size, determined by the number of eligible patients rather than by an a priori sample size calculation. Without a comparator group, like TURP, medical therapy, or watchful waiting, the observed changes in sexual function cannot be causally attributed to TPLA. Several alternative explanations cannot be excluded: (a) regression to the mean, although inclusion was based on urinary rather than sexual-function criteria; (b) indirect effects of marked urinary symptom relief on sexual function; (c) discontinuation of alpha-blockers or 5-ARIs after symptomatic improvement, which was not systematically documented and may have confounded the observed changes in MSHQ-EjD scores; and (d) attention and expectation effects inherent to an unblinded study with repeated assessments. Further limitations include the selection of only sexually active men with a stable partner (14 of 57 treated patients were excluded as not sexually active), which may have favored couples with better baseline sexual function and limits generalizability; a complete-case analysis, which may introduce bias if patients with less favorable outcomes are more likely to be lost to follow-up, although attrition was minimal (1 of 37 eligible patients); the performance of all procedures by a single experienced surgeon, which may limit generalizability to less experienced operators; the absence of objective confirmation of antegrade ejaculation and ejaculate volume; the large number of exploratory correlation analyses, in which the nominally significant associations did not survive adjustment for multiple comparisons and may represent false-positive findings; the inability of the inclusion criteria to fully exclude occult microscopic prostate cancer, together with a 12-month PSA follow-up that may be insufficient to detect slowly progressing tumors, and post-ablation changes that may complicate subsequent interpretation of PSA kinetics and mpMRI; and a 12-month follow-up insufficient to establish long-term durability. Moreover, partner-specific characteristics, including age, menopausal and hormonal status, gynecological conditions, relationship duration, relationship quality, comorbidities, and pre-existing sexual dysfunction, were not recorded owing to the retrospective design. As these factors are recognized determinants of female sexual function, they may have confounded the FSFI outcomes, and the partner data should therefore be regarded as descriptive and hypothesis-generating.

## 5. Conclusions

In this retrospective single-arm cohort, TPLA was followed by substantial relief of severe LUTS and preservation of erectile function. After a transient early decline, ejaculatory function and partner sexual function scores recovered and were slightly higher than baseline at 12 months. These later changes should be interpreted as associations rather than treatment effects. TPLA may be considered for sexually active men who prioritize preservation of erectile and ejaculatory function, and counseling should address the possibility of transient early sexual impairment. Controlled studies are required to establish whether TPLA itself improves ejaculatory or partner sexual function.

## Figures and Tables

**Table 1:** Baseline demographic and clinical characteristics of the study population (N = 36).

Variable	Value, Median (IQR)
Age, years	59.0 (9.75)
BMI, kg/m^2^	25.0 (4.00)
PSA, ng/mL	3.75 (1.89)
Prostate volume, mL	70.0 (19.50)
QoL score	4.0 (1.00)
Qmax, mL/s	7.0 (2.00)
PVR, mL	280.0 (167.50)
IPSS	31.0 (6.00)
IIEF-5	16.0 (5.00)
MSHQ-EjD	11.0 (3.00)
MSHQ-bother domain	3.0 (1.00)
Partner FSFI	23.0 (9.75)

BMI, body mass index; PSA, prostate-specific antigen; IPSS, International Prostate Symptom Score; QoL, quality of life; Qmax, maximum urinary flow rate; PVR, post-void residual volume; IIEF-5, International Index of Erectile Function-5; MSHQ-EjD, Male Sexual Health Questionnaire for Ejaculatory Dysfunction; FSFI, Female Sexual Function Index (partner-reported). IQR, interquartile range.

**Table 2:** Perioperative parameters.

Variable	Value, Median (IQR)
Procedure duration, min	27.5 (13.0)
Total laser energy delivered, J	7200 (3600)
Energy density, J/mL	81.35 (26.11)
Optical fibers used, *n*	2 (1.0) [range 1–2]
Intraoperative VAS pain score	3.0 (2.0)
Charlson Comorbidity Index	2.0 (2.0)

TPLA, transperineal laser ablation; VAS, visual analogue scale; IQR, interquartile range. Energy density was calculated as total laser energy delivered divided by baseline prostate volume.

## Data Availability

The data presented in this study are available on request from the corresponding author. The data are not publicly available due to ethical restrictions and patient privacy.
